# Maternal Prenatal Cannabis Use and Child Autism Spectrum Disorder

**DOI:** 10.1001/jamanetworkopen.2024.40301

**Published:** 2024-10-18

**Authors:** Lyndsay A. Avalos, Mahlet Shenkute, Stacey E. Alexeeff, Nina Oberman, Lisa A. Croen, Meghan Davignon, Sara R. Adams, Deborah Ansley, Carley Castellanos, Kelly C. Young-Wolff

**Affiliations:** 1 Division of Research, Kaiser Permanente Northern California, Pleasanton; 2 Bernard J. Tyson Kaiser Permanente School of Medicine, Pasadena, California; 3 Kaiser Permanente Roseville Medical Center, Pediatric Subspecialties; Regional Medical Director of Pediatric Developmental Disabilities, Roseville, California; 4 Regional Offices, Kaiser Permanente Northern California, Oakland; 5 Department of Psychiatry and Behavioral Sciences, University of California, San Francisco

## Abstract

**IMPORTANCE:**

Despite an increase in maternal prenatal cannabis use and associations with adverse neonatal outcomes, research on child neurodevelopmental outcomes is limited.

**OBJECTIVE:**

To evaluate the association between maternal cannabis use in early pregnancy and child autism spectrum disorder (ASD).

**DESIGN, SETTING, and PARTICIPANTS:**

This population-based retrospective birth cohort study included children born between 2011 and 2019 to pregnant Kaiser Permanente Northern California members screened for prenatal cannabis use during pregnancy. Statistical analysis was conducted February 2023 to March 2024.

**EXPOSURES:**

Maternal prenatal cannabis use was assessed at entrance to prenatal care (approximately 8- to 10-weeks’ gestation) via self-report and/or positive urine toxicology test. Use frequency was assessed.

**Main Outcomes and Measures:**

Child ASD was defined by *International Statistical Classification of Diseases and Related Health Problems, Tenth Revision *(*ICD-10*) diagnosis codes ascertained from the electronic health record. Associations between maternal prenatal cannabis use and child ASD were modeled using Cox proportional hazards regression adjusted for maternal sociodemographic, other substance use and disorders, prenatal care initiation, comorbidities, and clustering among maternal siblings.

**RESULTS:**

The study cohort included 178 948 singleton pregnancies among 146 296 unique pregnant individuals, including 48 880 (27.3%) Asian or Pacific Islander, 42 799 (23.9%) Hispanic, 9742 (5.4%) non-Hispanic Black, and 70 733 (39.5%) non-Hispanic White pregnancies. The median (IQR) maternal age at pregnancy onset was 31 (6) years; 8486 (4.7%) screened positive for cannabis use, 7054 (3.9%) via urine toxicology testing and 3662 (2.0%) by self-report. In the total study population, the frequency of self-reported use was monthly or less for 2003 pregnancies (1.1%), weekly for 918 pregnancies (0.5%), daily for 741 pregnancies (0.4%), and unknown for 4824 pregnancies (2.7%). ASD was diagnosed in 3.6% of children. After adjustment for maternal characteristics, maternal prenatal cannabis use was not associated with child ASD (hazard ratio [HR], 1.05; 95% CI, 0.84-1.32). When self-reported frequency of use was assessed, no statistically significant associations were observed after confounder adjustment. No sex-specific associations were documented (males: HR, 1.01; 95% CI, 0.77-1.32; and females: HR, 1.19; 95% CI, 0.77-1.85).

**CONCLUSIONS and Relevance:**

In this cohort study, maternal cannabis use assessed in early pregnancy was not associated with child ASD. Additional studies are needed to evaluate different patterns of use throughout pregnancy. Given the known adverse neonatal health effects of maternal prenatal cannabis use, clinicians should follow national guidelines and advise against use.

## Key Points

**Question:**

Is maternal cannabis use during early pregnancy associated with risk of child autism spectrum disorder (ASD)?

**Findings:**

In this cohort study of 178 948 mother-child dyads, maternal prenatal cannabis use during early pregnancy was not associated with child ASD.

**Meaning:**

These findings suggest that maternal cannabis use during early pregnancy was not associated with child ASD, but additional research should be conducted to replicate these findings.

## Teaser

This cohort study examines the association of maternal prenatal cannabis use in early pregnancy and child autism spectrum disorder.

## Introduction

Maternal prenatal cannabis use has been steadily rising across the United States.^1,2^ With the increase in cannabis legalization for medical and recreational use,^3^ acceptance and accessibility has also been increasing.^4^ Among pregnant individuals, there is a growing perception that cannabis is a lower risk alternative to some prescription medications during pregnancy.^5^ However, cannabinoids can freely cross the placenta and enter the fetal blood stream with potential to disrupt fetal neurodevelopment. Despite an increasing body of research documenting adverse infant health effects, data on the long-term child neurodevelopmental outcomes of maternal prenatal cannabis use are limited.^6^

To date, studies evaluating the association between maternal prenatal cannabis use and child autism spectrum disorders (ASD) or ASD-related traits have reported conflicting results.^7,8,9,10^ A population-based retrospective cohort study conducted in Canada documented an increased risk of ASD diagnoses associated with maternal self-reported prenatal cannabis use.^7^ In contrast, a large multisite case-control study in the US reported no association between peripregnancy (before and during pregnancy and while breastfeeding) cannabis use and ASD diagnoses.^8^ Two additional cohort studies evaluating maternal-reported autism-related traits, as opposed to diagnoses, reported no association with prenatal cannabis use.^9,10^ Cannabis exposure assessment in each study was limited to self-report of any use during pregnancy and did not include information on the frequency of use. Additionally, none of the studies evaluated sex-specific associations.

The current study used data from Kaiser Permanente Northern California’s (KPNC) large integrated health care delivery system, which universally screens pregnant individuals for prenatal cannabis use via self-report and a urine toxicology test and uses evidence-based practices to screen and assess children for autism spectrum disorders (ASD). We aimed to evaluate the association between maternal prenatal cannabis use assessed in early pregnancy and ASD in children aged 12 years and younger.

## Methods

This cohort study was approved by the institutional review board at KPNC and the State of California with a waiver of consent. This study followed the Strengthening the Reporting of Observational Studies in Epidemiology (STROBE) reporting guideline.

### Setting

KPNC is an integrated health care delivery system that provides health care to more than 4.6 million members. KPNC health plan members are covered by employer-sponsored insurance plans, the California insurance exchange, Medicare, and Medicaid. Information on diagnoses, hospitalizations, outpatient visits, and prescribed medications are maintained within administrative and electronic health records (EHR). Coverage is provided for approximately 40% of northern California and membership is similar to the population living in the geographic area.^11^ As part of standard prenatal care, pregnant KPNC patients are universally screened for prenatal substance use by both self-report (via a self-administered questionnaire) and a urine toxicology test to which they consent. Pediatric care includes universal autism screening using the Survey of Well-Being in Young Children, Parents Observation of Social Interactions at the 18- and 24- months well-child visits as well as routine surveillance for developmental concerns at all well-child visits.

### Cohort

We conducted a population-based retrospective cohort study of pregnant individuals and their singleton children born between January 1, 2011, and December 31, 2019, at a KPNC facility. All pregnancies of individuals with more than 1 pregnancy episode during the study period were included. Additional inclusion criteria included continuous KPNC health plan membership of the mother 1 year prior to pregnancy onset through delivery allowing for 90-day gaps in membership, maternal attendance at 1 or more KPNC prenatal care visits, and a response to the self-report question about cannabis use since pregnancy and a prenatal urine toxicology test for tetrahydrocannabinol (THC). Exclusion criteria included evidence of a maternal prenatal prescription fill for a teratogenic, antineoplastic, or antiepileptic drug anytime during pregnancy. Infants who died prior to age 12 months without KPNC health plan enrollment in the month infant turned 1 year. Additionally, maternal-infant dyads with missing parity (0.02%), or missing NDI data (0.02%) were excluded (eFigure 1 in Supplement 1). Children were followed until the end of the study period (December 31, 2022) or until they reached age 12 years. Data were obtained from KPNC’s administrative and EHR databases (including KPNC’s Autism Registry) and from California State Birth Certificates.

### Measures

#### Exposure

The primary exposure was prenatal cannabis use in early pregnancy assessed at entrance to prenatal care (typically at approximately 8- to 10- weeks’ gestation) based on self-reported maternal cannabis use and/or a positive urine toxicology test for THC. Among those with self-reported data, frequency of cannabis use during pregnancy was categorized as never, monthly or less, weekly, daily, or unknown frequency (no self-reported use and a positive toxicology result for THC).

### Outcome

#### Autism Spectrum Disorder (ASD)

Child medical record numbers obtained from the EHR were linked to the KPNC Autism Registry to identify children diagnosed with ASD. At least 1 ASD diagnosis by a KPNC ASD center or at least 2 ASD diagnoses on separate dates recorded by a clinician not affiliated with a KPNC ASD center were required to define our outcome. In this cohort, the majority of children with ASD (86.9%) were diagnosed at a KPNC ASD evaluation center by a multidisciplinary team using a standardized protocol, including the Autism Diagnostic Observation Schedule Generic (ADOS-G),^12^ the gold standard clinical assessment tool. The psychologists doing the evaluations at the KPNC ASD centers are ASD experts, having been trained on the ADOS-G as well as other standardized assessment tools used in a comprehensive ASD evaluation (eg, Vineland, Mullen). The use of at least 2 ASD diagnoses has been previously validated for use in large integrated health care settings to identify ASD patients for population-based research.^13^ *International Classification of Diseases, Ninth Revision *(*ICD-9*) codes (299.0, 299.8, and 299.9) and *International Statistical Classification of Diseases and Related Health Problems, Tenth Revision (ICD-10)* codes (F84.0, F84.5, F84.8, and F84.9) were applied to define ASD diagnoses.

### Covariates

Maternal sociodemographic covariates were ascertained from the EHR and supplemented with birth certificate data. Self-reported race and ethnicity was categorized as Asian or Pacific Islander, non-Hispanic Black, Hispanic, non-Hispanic White, other or unknown. Other includes American Indian, Alaska Native, and multiracial individuals.

Age at pregnancy onset (younger than 18 years, 18 to 24 years, 25 to 30 years, 31 to 35 years, and 36 years or older), insurance type (Medicaid vs other), parity (0, 1, 2 or more), and education (high school or less, some college, college graduate, graduate school, and unknown) were included. Neighborhood Deprivation Index (NDI) categorized into quartiles, and year of birth was included as an integer variable spanning the study period (2011 through 2019). Other noncannabis prenatal substance use included alcohol, nicotine, opioids, stimulants, and anxiety or sleep medications evaluated at entrance into prenatal care by self-report and urine toxicology (eAppendix in Supplement 1).

Prenatal care initiation was assessed using a 1-factor Kotelchuck Month of Initiation Index and categorized as adequate plus (month 1 to 2), adequate (month 3 to 4), intermediate (month 5 to 6) and inadequate (month 7 or more).^14^ Maternal comorbidities defined by *ICD-9* and *ICD-10* codes included preexisting diabetes (in the 2 years before pregnancy onset through the first prenatal visit), preexisting asthma, mood or anxiety disorders, other psychiatric disorders, chronic pain, noncannabis substance use disorders (diagnosed in the year prior to pregnancy onset through the first prenatal visit), and nausea or vomiting during pregnancy (diagnosed between pregnancy onset and first prenatal care visit). Antidepressant medication use was defined as a prescription dispensing between pregnancy onset and first prenatal visit.

### Statistical Analysis

Cox proportional hazards regression models were fit to estimate the association between maternal prenatal cannabis use and ASD. Age of the child (in months) was used as the time scale with follow-up time starting at 12 months of age up to a maximum age of follow up (143 months). Attendance of routine well-child visits was required to remain in follow-up. The time of outcome was defined as the age at first ASD diagnosis. Children without the outcome were right censored based on the minimum of the following 3 criteria: end of KPNC health plan membership (a gap of more than 3 months of enrollment), a missed well-child visit, or death. Children who were not censored were followed to the end of the study (December 31, 2022). Inverse probability of censoring weights (IPCW) were applied to account for the potential impact of informative censoring since prior research on this cohort found a lower attendance of well-child visits among children of cannabis users vs nonusers.^15,16^ Time varying stabilized weights were generated for 6 censoring time intervals based on the recommended well-child visit schedule (months 12 to 27, 28 to 41, 42 to 65, 66 to 77, 78 to 101, and 102 to 143). Marginal Cox models with a cluster term at the maternal level and robust standard errors were used to account for associated observations (ie, multiple singleton pregnancies nested within individuals).

The association between maternal prenatal cannabis use and ASD in the overall cohort was assessed for any prenatal cannabis use (yes vs no) and the frequency of cannabis use (never, monthly or less, weekly, daily, or unknown frequency). For all analyses performed (main and sensitivity), models were fit sequentially to examine the degree of confounding by certain sets of confounders. Factors that occurred after the exposure assessment (eg, preterm birth) that would be considered mediators were not included in any models. Model 1 did not include any covariates. Model 2 was adjusted for maternal sociodemographic characteristics (age, race or ethnicity, education, parity, NDI, Medicaid, and birth year). Model 3 was additionally adjusted for other noncannabis substance use during pregnancy (alcohol, opiates, stimulants, nicotine, or anxiety and sleep medication). Model 4 was further adjusted for adequacy of prenatal care. Model 5 additionally included maternal comorbidities (asthma, diabetes, nausea or vomiting during pregnancy, mood or anxiety disorders, other psychiatric disorders, substance use disorders, chronic pain) and antidepressant use during pregnancy. Missing data for maternal race and ethnicity, maternal education, and frequency of maternal cannabis use were modeled using a missing category. All models were adjusted for child’s age (by using age as the time scale), probability of censoring (IPCW), and correlation among maternal siblings (maternal cluster).

Additional analyses were conducted with any maternal prenatal cannabis use determined by self-report or urine toxicology test. Sensitivity analyses evaluating any prenatal cannabis use and frequency of cannabis use were conducted after excluding pregnancies with any evidence of noncannabis prenatal substance use. Given the differential prevalence of ASD between males and females, we also evaluated the association between prenatal cannabis exposure and ASD stratified by child sex. Analyses were conducted between February 2023 to March 2024 and performed using SAS version 9.4 (SAS Institute) and R version 4.0.2 (R Project for Statistical Computing), and 2-sided *P* < .05 were considered statistically significant.

## Results

**Figure 1.**
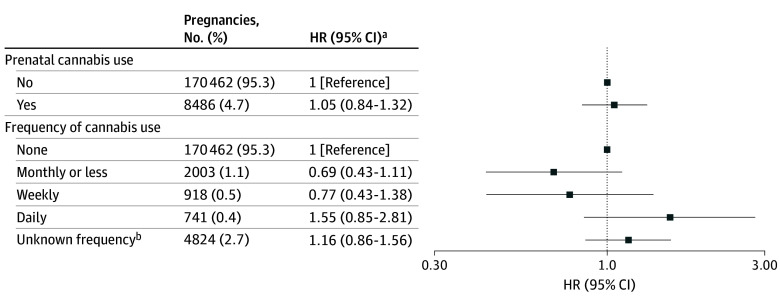
Association of Maternal Prenatal Cannabis Use and Frequency of Cannabis Use With Autism Spectrum Disorder (N = 178 948 Parent-Infant Dyads) ^a^Adjusted for maternal demographics, other noncannabis substance use (alcohol, nicotine, opioids, anxiety or sleep medication, and stimulants), prenatal care initiation (Kotelchuck month of initiation index), medical and mental health comorbidities (asthma, diabetes, nausea or vomiting during pregnancy, mood or anxiety disorders, other psychiatric disorders, substance use disorders, antidepressant use, chronic pain). ^b^Unknown frequency includes pregnancies that self-reported no cannabis use but had a positive urine toxicology result for tetrahydrocannabinol.

The study cohort included 178 948 singleton pregnancies (146 296 unique pregnant individuals) and included 48 880 (27.3%) pregnancies among Asian or Pacific Islander individuals, 42 799 (23.9%) among Hispanic individuals, 9742 (5.4%) among non-Hispanic Black individuals, and 70 733 (39.5%) among non-Hispanic White individuals (Table 1). The median (IQR) maternal age at pregnancy onset was 31 (6) years. Of the pregnancies, 8225 (4.6%) were insured by Medicaid, and 25 427 (14.2%) had a high school or less education level. A total of 8486 pregnancies (4.7%) screened positive for cannabis (3662 [2.0%] by self-report and 7054 [3.9%] by urine toxicology testing). In the total study population, the frequency of self-reported use was monthly or less for 2003 pregnancies (1.1%), weekly for 918 pregnancies (0.5%), daily for 741 pregnancies (0.4%), and unknown (ie, positive toxicology, but no self-reported use) for 4824 pregnancies (2.7%) (Figure 1). The median (IQR) gestational age at prenatal substance use screening was 8 (2.6) weeks.

**Table 1.** Characteristics of 178 948 Pregnancies at Kaiser Permanente Northern California, Overall and by Any Maternal Prenatal Cannabis Use

Characteristics	Pregnancies, No. (%)		
	Total^a^	Any maternal prenatal cannabis use^b^	
		Yes	No
Total	178 948	8486	170 462
Maternal sociodemographic characteristics			
Race and ethnicity			
Asian or Pacific Islander	48 880 (27.3)	545 (6.4)	48 335 (28.4)
Hispanic	42 799 (23.9)	2255 (26.6)	40 544 (23.8)
Non-Hispanic Black	9742 (5.4)	1833 (21.6)	7909 (4.6)
Non-Hispanic White	70 733 (39.5)	3326 (39.2)	67 407 (39.5)
Other or unknown^c^	6794 (3.8)	527 (6.2)	6267 (3.7)
Age category, y			
<18	1211 (0.7)	245 (2.9)	966 (0.6)
18-24	17 975 (10.0)	2797 (33.0)	15 178 (8.9)
25-30	58 780 (32.8)	2644 (31.2)	56 136 (32.9)
31-35	67 629 (37.8)	1965 (23.2)	65 664 (38.5)
≥36	33 353 (18.6)	835 (9.8)	32 518 (19.1)
Parity category			
0	73 479 (41.1)	4667 (55.0)	68 812 (40.4)
1	66 946 (37.4)	2320 (27.3)	64 626 (37.9)
≥2	38 523 (21.5)	1499 (17.7)	37 024 (21.7)
Insurance type			
Medicaid	8225 (4.6)	1600 (18.9)	6625 (3.9)
Other	170 723 (95.4)	6886 (81.1)	163 837 (96.1)
Education level			
High school or less	25 427 (14.2)	2666 (31.4)	22 761 (13.4)
Some college	50 796 (28.4)	3704 (43.6)	47 092 (27.6)
College graduate	60 564 (33.8)	1384 (16.3)	59 180 (34.7)
Graduate school	38 353 (21.4)	516 (6.1)	37 837 (22.2)
Unknown^d^	3808 (2.1)	216 (2.5)	3592 (2.1)
Neighborhood Deprivation Index			
Q1, least deprived	44 744 (25.0)	1112 (13.1)	43 632 (25.6)
Q2	44 733 (25.0)	1635 (19.3)	43 098 (25.3)
Q3	44 737 (25.0)	2274 (26.8)	42 463 (24.9)
Q4, most deprived	44 734 (25.0)	3465 (40.8)	41 269 (24.2)
Year of birth			
2011	17 349 (9.7)	685 (8.1)	16 664 (9.8)
2012	18 020 (10.1)	719 (8.5)	17 301 (10.1)
2013	18 238 (10.2)	718 (8.5)	17 520 (10.3)
2014	18 931 (10.6)	819 (9.7)	18 112 (10.6)
2015	19 172 (10.7)	842 (9.9)	18 330 (10.8)
2016	20 341 (11.4)	912 (10.7)	19 429 (11.4)
2017	21 031 (11.8)	1050 (12.4)	19 981 (11.7)
2018	22 429 (12.5)	1330 (15.7)	21 099 (12.4)
2019	23 437 (13.1)	1411 (16.6)	22 026 (12.9)
Noncannabis prenatal substance use^e^			
Alcohol use	17 101 (9.6)	1809 (21.3)	15 292 (9.0)
Nicotine use	6785 (3.8)	1912 (22.5)	4873 (2.9)
Anxiety or sleep medication use	5162 (2.9)	594 (7.0)	4568 (2.7)
Stimulant use	1097 (0.6)	265 (3.1)	832 (0.5)
Opioid use	4603 (2.6)	585 (6.9)	4018 (2.4)
Prenatal care initiation^f^			
Adequate plus (month 1-2)	121 765 (68.0)	5466 (64.4)	116 299 (68.2)
Adequate (month 3-4)	54 625 (30.5)	2743 (32.3)	51 882 (30.4)
Intermediate (month 5-6)	1892 (1.1)	192 (2.3)	1700 (1.0)
Inadequate (month ≥7)	666 (0.4)	85 (1.0)	581 (0.3)
Maternal comorbidities			
Preexisting asthma	18 144 (10.1)	1529 (18.0)	16 615 (9.7)
Preexisting chronic pain	6012 (3.4)	587 (6.9)	5425 (3.2)
Preexisting diabetes (Type 1 or 2)	2515 (1.4)	104 (1.2)	2411 (1.4)
Preexisting mood or anxiety disorder	20 417 (11.4)	1992 (23.5)	18 425 (10.8)
Preexisting other psychiatric disorder	4264 (2.4)	556 (6.6)	3708 (2.2)
Preexisting substance use disorder^g^	5532 (3.1)	1409 (16.6)	4123 (2.4)
Antidepressant use^h^	8035 (4.5)	772 (9.1)	7263 (4.3)
Nausea or vomiting^i^	19 123 (10.7)	1946 (22.9)	17 177 (10.1)
Offspring sex			
Female	87 072 (48.7)	4145 (48.8)	82 927 (48.6)
Male	91 876 (51.3)	4341 (51.2)	87 535 (51.4)

^a^ The cohort consisted of 178 948 pregnancies among 146 296 unique individuals.

^b^ Any prenatal cannabis use was determined by self-report and/or urine toxicology testing.

^c^ Other race and ethnicity includes American Indian, Alaska Native, and multiracial individuals.

^d^ Missing maternal education level from both birth certificate and self-report.

^e^ Assessed at entrance to prenatal care or prescription filled during pregnancy through date of first prenatal visit.

^f^ Adequacy of prenatal care was entry month at prenatal care initiation, derived from the first prenatal office visit as part of the Kotelchuck index.

^g^ Preexisting substance use disorder excludes cannabis-related substance use disorders.

^h^ Antidepressant dispensing between pregnancy onset and first prenatal visit.

^i^ Nausea or vomiting diagnosis between pregnancy onset and first prenatal office visit.

The cohort of children included 91 876 males (51.3%) and 87 072 females (48.7%). The median (IQR) length of follow-up was 3.7 (3.0) years and the prevalence of ASD in our cohort was 6463 (3.6%) diagnosed at median (IQR) age of 3 (2) years (Table 2). Among children with ASD, 1453 (22.5%) were females and 5010 (77.5%) were males.

**Table 2.** Associations Between Maternal Prenatal Cannabis Use and Autism Spectrum Disorder (ASD) Among a Kaiser Permanente Northern California Birth Cohort, N = 178 948 Parent-Infant Dyads

Outcome	ASD cases, No. (row %)^a^	Hazard ratio (95% CI)				
		Model 1^b^	Model 2^c^	Model 3^d^	Model 4^e^	Model 5^f^
Any cannabis use						
No	6092 (3.6)	1 [Reference]	1 [Reference]	1 [Reference]	1 [Reference]	1 [Reference]
Yes	371 (4.4)	1.25 (1.10-1.42)^g^	1.10 (0.87-1.38)	1.09 (0.87-1.37)	1.09 (0.87-1.37)	1.05 (0.84-1.32)
Frequency of cannabis use						
None	6092 (3.6)	1 [Reference]	1 [Reference]	1 [Reference]	1 [Reference]	1 [Reference]
Monthly or less	70 (3.5)	0.79 (0.50-1.24)	0.69 (0.44-1.10)	0.71 (0.45-1.14)	0.71 (0.45-1.14)	0.69 (0.43-1.11)
Weekly	43 (4.7)	0.96 (0.53-1.72)	0.80 (0.45-1.44)	0.80 (0.45-1.45)	0.80 (0.45-1.45)	0.77 (0.43-1.38)
Daily	36 (4.9)	2.12 (1.19-3.78)^g^	1.67 (0.93-3.01)	1.68 (0.93-3.03)	1.67 (0.93-3.02)	1.55 (0.85-2.81)
Unknown frequency^h^	222 (4.6)	1.42 (1.06-1.91)^g^	1.22 (0.91-1.64)	1.19 (0.89-1.60)	1.20 (0.89-1.60)	1.16 (0.86-1.56)

^a^ The prevalence of Autism Spectrum Disorder in overall cohort.

^b^ Unadjusted Cox proportional hazards model with inverse-probability of censoring weights applied and maternal-level cluster term.

^c^ Adjusted for maternal demographics (age at pregnancy onset, race and ethnicity, education, neighborhood deprivation index, parity, Medicaid status, infant birth year).

^d^ Additionally adjusted for other noncannabis substance exposure (alcohol, nicotine, opioids, anxiety or sleep medication, stimulants).

^e^ Additionally adjusted for month of prenatal care initiation.

^f^ Additionally adjusted for maternal medical and mental health comorbidities (asthma, diabetes mellitus, nausea or vomiting during pregnancy, mood or anxiety disorders, other psychiatric disorders, substance use disorders, antidepressant use, chronic pain).

^g^ Denote statistical significance at the *P* < .05 level.

^h^ Unknown frequency includes pregnancies that self-reported no cannabis use but tested positive on urine toxicology.

**Figure 2.**
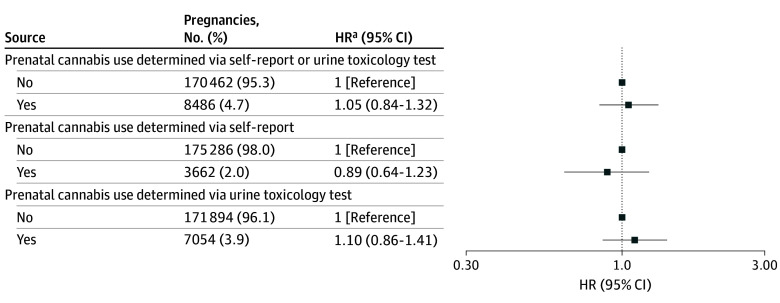
Comparing Maternal Prenatal Cannabis Use Ascertained From Self-Report vs Maternal Prenatal Cannabis Use Ascertained From Urine Toxicology Test (N = 178 948 Parent-Infant Dyads) HR indicates hazard ratio. ^a^Adjusted for demographics, other noncannabis substance use (alcohol, nicotine, opioids, anxiety or sleep medication, and stimulants), prenatal care initiation (Kotelchuck month of initiation index), medical and mental health comorbidities (asthma, diabetes, nausea or vomiting during pregnancy, mood or anxiety disorders, other psychiatric disorders, substance use disorders, antidepressant use, chronic pain).

Any maternal prenatal cannabis use early in pregnancy was associated with an increased risk of child ASD in model 1 (hazard ration [HR], 1.25; 95% CI, 1.10-1.42) (Table 2), but the effect estimate was attenuated and not statistically significant after adjustment for maternal sociodemographic characteristics (model 2: HR, 1.10; 95% CI, 0.87-1.38). The fully adjusted model 5 demonstrated similarly attenuated and nonstatistically significant results (HR, 1.05; 95% CI, 0.84-1.32) (Table 2 and Figure 1). In sensitivity analyses, no association with child ASD was documented when prenatal cannabis was defined by self-report (HR, 0.89; 95% CI, 0.64-1.23) or by a toxicology test (HR, 1.10; 95% CI, 0.86-1.41) (Figure 2).

When assessing self-reported frequency of use, there was a statistically significant increased risk of child ASD associated with daily use in model 1 (HR, 2.12; 95% CI, 1.19-3.78) (Table 2), but after adjustment for maternal sociodemographic characteristics the risk was attenuated and no longer statistically significant (model 2: HR, 1.67; 95% CI, 0.93-3.01) (Table 2). Adjustment for all potential confounders (model 5) similarly produced elevated point estimates that did not reach statistical significance (HR, 1.55; 95% CI, 0.85-2.81) (Table 2 and Figure 1). There was no association with ASD for monthly or less (HR, 0.69; 95% CI, 0.44-1.10) or weekly (HR, 0.80; 95% CI, 0.45-1.44) use compared with no use in the minimally adjusted model 2 (HR, 0.69; 95% CI, 0.43-1.11) and findings were similar in the fully adjusted model 5 (HR, 0.77; 95% CI, 0.43-1.38) (Table 2 and Figure 1). Unknown frequency of use with a positive toxicology test was significantly associated with child ASD (HR, 1.42; 95% CI, 1.06-1.91) in model 1, but results were attenuated and no longer statistically significant after adjustment for maternal sociodemographic characteristics (model 2: HR, 1.22; 95% CI, 0.91-1.64). The fully adjusted model 5 produced similar attenuated and nonstatistically significant results (HR, 1.16; 95% CI, 0.86-1.56) (Table 2 and Figure 1).

When stratified by child sex and after confounder adjustment, maternal prenatal cannabis use was not statistically associated with ASD among males (HR, 1.01; 95% CI, 0.77-1.32) or females (HR, 1.19; 95% CI, 0.77-1.85) (eTable in Supplement 1). No statistically significant associations were noted for the frequency of prenatal cannabis use and ASD risk for either sex (eTable in Supplement 1).

Sensitivity analysis excluding individuals with prenatal noncannabis substance exposure demonstrated similar findings to the main analysis with no significant association between any maternal prenatal cannabis use or frequency of cannabis use and child ASD (eFigure 2 in Supplement 1).

## Discussion

In this large, population-based birth cohort study with one of the largest numbers of children born to individuals with maternal prenatal cannabis use (8486 children) to date, we found no association between prenatal cannabis use in early pregnancy and child ASD. However, although findings did not reach the level of statistical significance after adjustment for sociodemographic characteristics, our data suggests there may be an association with higher frequency use, highlighting the need for more research. Study findings remained relatively stable when maternal prenatal cannabis use was defined by self-report, by a urine toxicology test, and after excluding pregnancies with other noncannabis prenatal substance use. This study adds to the nascent literature on longer-term child developmental outcomes associated with maternal prenatal cannabis use by using a well-established population in which adverse neonatal outcomes associated with prenatal cannabis use has been previously reported.^17^

The finding of a lack of an association between any maternal prenatal cannabis use and child ASD is similar to a study using ASD diagnoses (adjusted odds ratio, 0.89; 95% CI,0.62-1.27)^8^ and both studies evaluating ASD-related traits.^9,10^ Our findings are in contrast with the positive association (aHR, 1.51; 95% CI, 1.17-1.96) noted by Corsi et al^7^ which relied on self-report of any maternal prenatal cannabis use and reported a lower prevalence of use (0.6%)^7^ compared with the prevalence in other studies that did not find an association (4.3%^8^ and 10.2%^10^).

Cannabinoids, including THC, have been demonstrated to impact the placenta adversely.^18,19,20,21^ The placenta produces neurotransmitters that may affect fetal brain development, and recent research has linked placental dysfunction with adverse child neurodevelopmental outcomes.^22,23^ Animal models have demonstrated neuroteratogenic effects of prenatal THC exposure, resulting in long-lasting neurodevelopmental and behavioral abnormalities in the offspring, with some studies noting differential effects for female and male offspring.^24,25,26^ Epidemiologic research has also documented changes to child brain structure (eg, cortical thinning in the superior frontal and superior parietal cortices) and function (eg, neurophysiological processing during executive functioning), with prenatal cannabis exposure.^27,28,29^ Yet, the findings on neurobehavioral and cognitive outcomes have been inconclusive.^30,31^ While we did not find an increase in ASD associated with maternal prenatal cannabis use overall, our results suggest there may be an elevated risk for females and for daily use, warranting additional research. Given evidence that prenatal cannabis use is associated with an increased risk of adverse maternal, fetal, and neonatal health-effects^17,32,33,34,35^ and increased child psychopathology^36,37^ pregnant individuals should discontinue cannabis use as recommended by American College of Obstetrics and Gynecology and American Academy of Pediatrics.^38^

This study has several important strengths. To our knowledge, this contemporary cohort inclusive of recent trends in cannabis products and potency represents the largest number of pregnancies with maternal prenatal cannabis use studied. The cohort of parent-child dyads was ascertained prospectively with screening for maternal prenatal cannabis use at entrance to prenatal care via urine toxicology testing and self-report, and children routinely screened at multiple time points for ASD, thus reducing the potential for recall bias and misclassification. We captured many potential confounders through the EHR to increase methodologic rigor and reduce potential bias and residual confounding. We conducted analyses to address variance in exposure classification by self-report and urine toxicology and confounding by co-occurrence of maternal prenatal noncannabis substance use which all produced similar results. Additionally, this study is among the first to assess the frequency of maternal prenatal cannabis use and sex-related associations with child neurodevelopment. Finally, the study sample was racially, ethnically, and geographically diverse with high generalizability.

### Limitations

This study has limitations. Maternal prenatal cannabis use was measured once in early pregnancy. However, research indicates the prevalence of cannabis use decreases by nearly half over the course of pregnancy (5.3% in the first trimester to 2.5% in the second and third trimester).^2^ Furthermore, it is well-established that the first trimester of pregnancy is a critical period for brain development signifying the importance of early pregnancy exposure assessment.^39^ Additionally, we do not have information on the route of administration (eg, vaping, smoking), the concentration of THC, which has increased throughout the study period, duration of use, or postnatal exposure through breastmilk. Given that cannabinoids can cross the placenta freely, future research on other cannabinoids (eg, cannabidiol [CBD], delta-8 THC) is important as their use continues to rise.^40^ Children of individuals who used cannabis during pregnancy were less likely to attend well-child visits in the first 3 years of life,^16^ and more likely to end their KPNC insurance coverage after birth, reducing the opportunity for evaluation and diagnosis of ASD. We used rigorous statistical techniques, including censoring and applying IPCW, but it may not have adequately addressed differential follow-up. It is possible that some children who were not diagnosed with ASD by the end of study follow-up will be diagnosed with ASD later in childhood. However, our cohort study design and Cox proportional hazards modeling appropriately accounts for varying lengths of follow-up and ensures that all model comparisons are conducted within risk sets of children who are the same age. Finally, this study was limited to individuals with insurance and may not generalize to uninsured populations. However, we note the US population prevalence estimates of maternal prenatal cannabis use (3% to 7%)^2,41^ and ASD (2.3% to 4.5%)^42^ are similar to that documented in the current study.

## Conclusions

In this study, maternal prenatal cannabis use was not associated with child ASD after adjusting for potential confounders, including sociodemographic characteristics, other noncannabis substance use and maternal comorbidities. Future research should further explore patterns of maternal prenatal cannabis use throughout pregnancy and sex-specific associations. Additionally, future investigations should consider the timing, duration, and cannabis potency. Despite these findings, pregnant individuals and those considering pregnancy should be educated on the known adverse fetal and neonatal health-effects of maternal prenatal cannabis use.^34,35,43,44^

## Notes

Supplement 1. **eFigure 1. **STROBE Diagram of Study Inclusion and Exclusion of Infants Born to Mothers Screened for Substance Use During Pregnancy at Kaiser Permanente Northern California from January 1, 2011 to December 31, 2019 **eFigure 2. **Adjusted Hazard Ratios for Associations Between Maternal Prenatal Cannabis Use and ASD, Excluding Pregnancies Exposed To Noncannabis Substances **eAppendix**. Ascertainment of Maternal Prenatal Substance Use **eTable. **Adjusted Hazard Ratios for Associations Between Maternal Prenatal Cannabis Use and ASD, Stratified by Infant Sex

Supplement 2.
Data Sharing Statement
