# Supplementary material for: Maternal Prenatal Cannabis Use and Child Autism Spectrum Disorder
Source: JAMA Netw Open. 2024 Oct 18;7(10):e2440301. doi: 10.1001/jamanetworkopen.2024.40301 (PMC11581557; doi:10.1001/jamanetworkopen.2024.40301)
Supplement: Supplement 1. — eFigure 1. STROBE Diagram of Study Inclusion and Exclusion of Infants Born to Mothers Screened for Substance Use During Pregnancy at Kaiser Permanente Northern California from January 1, 2011 to December 31, 2019 eFigure 2. Adjusted Hazard Ratios for Associations Between Maternal Prenatal Cannabis Use and ASD, Excluding Pregnancies Exposed To Noncannabis Substances eAppendix. Ascertainment of Maternal Prenatal Substance Use eTable. Adjusted Hazard Ratios for Associations Between Maternal Prenatal Cannabis Use and ASD, Stratified by Infant Sex [file jamanetwopen-e2440301-s001.pdf]

Supplemental Online Content

Avalos LA, Shenkute M, Alexeef SE, et al. Maternal prenatal cannabis use and child autism spectrum disorder. *JAMA Netw Open*. 2024;7(10):e2440301.
doi:10.1001/jamanetworkopen.2024.40301

eFigure 1. STROBE Diagram of Study Inclusion and Exclusion of Infants Born to Mothers Screened for Substance Use During Pregnancy at Kaiser Permanente Northern California from January 1, 2011 to December 31, 2019
eFigure 2. Adjusted Hazard Ratios for Associations Between Maternal Prenatal Cannabis Use and ASD, Excluding Pregnancies Exposed To Non-Cannabis Substances
eAppendix 1. Ascertainment of Maternal Prenatal Substance Use
eTable. Adjusted Hazard Ratios for Associations Between Maternal Prenatal Cannabis Use and ASD, Stratified by Infant Sex

This supplemental material has been provided by the authors to give readers additional information about their work.

© 2024 Avalos LA et al. *JAMA Network Open.*

eFigure 1. STROBE Diagram of Study Inclusion and Exclusion of Infants Born to Mothers Screened for Substance Use During Pregnancy at Kaiser Permanente

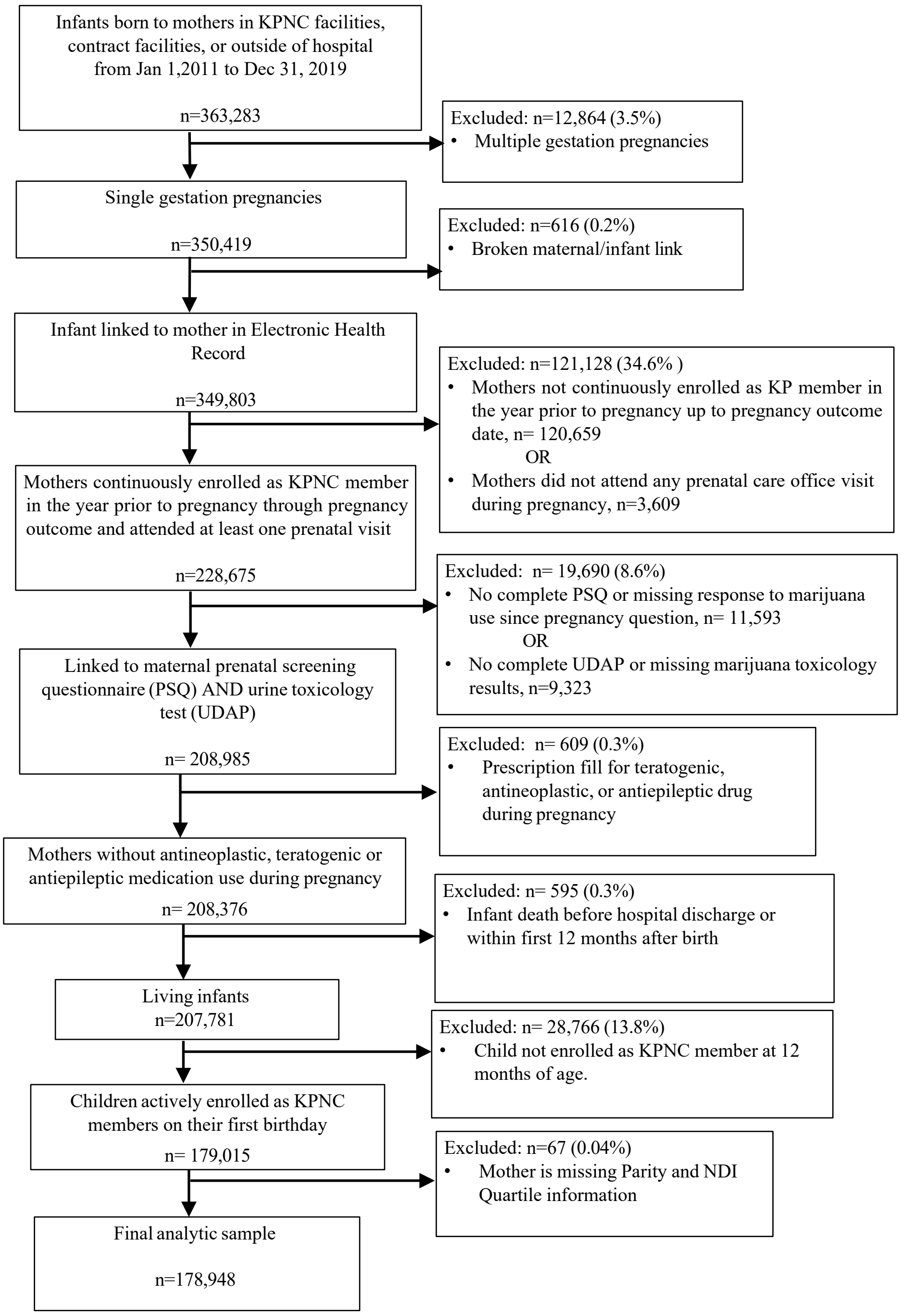

Northern California (KPNC) from January 1, 2011 to December 31, 2019

© 2024 Avalos LA et al. *JAMA Network Open.*

eFigure 2. Adjusted Hazard Ratios for Associations Between Maternal Prenatal Cannabis Use and ASD, Excluding Pregnancies Exposed To Non-Cannabis Substances

	Pregnancies, n(%)	HR (95% CI)[a]
Prenatal Cannabis Use		
No	144,558 (96.9)	1.00 (Ref)
Yes	4,656 (3.1)	1.00 (0.74– 1.35)
Frequency of Cannabis Use		
None	144,558 (96.9)	1.00 (Ref)
Monthly or less	715 (0.5)	0.63 (0.29– 1.35)
Weekly	298 (0.2)	0.48 (0.14– 1.69)
Daily	231 (0.2)	0.55 (0.16– 1.90)
Unknown frequency[b]	3,412 (2.3)	1.16 (0.83– 1.61)

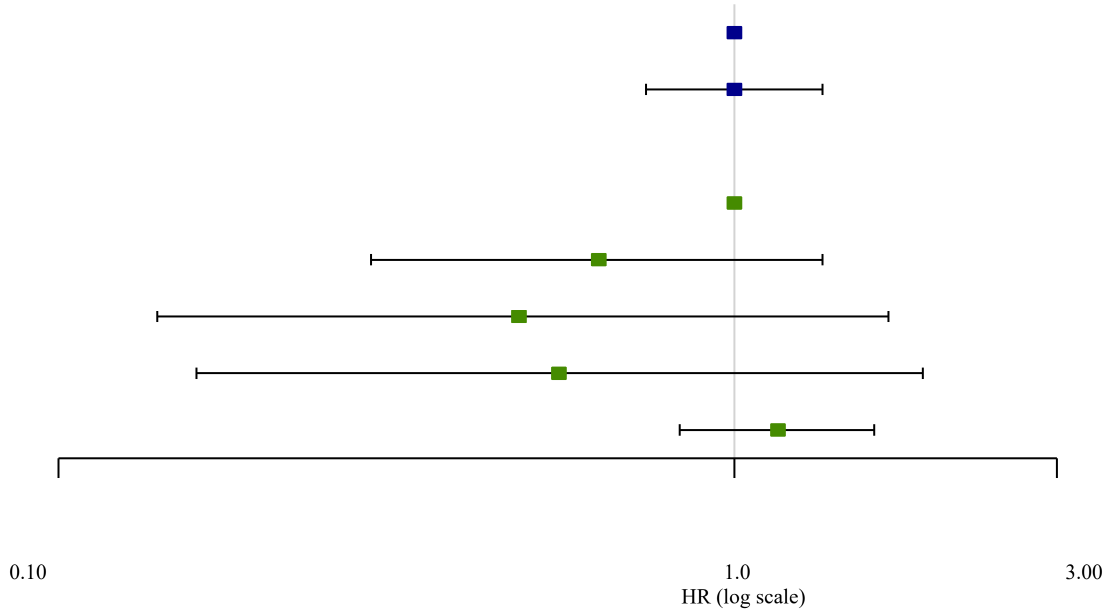

[a]HR: Adjusted for sociodemographics characterstics, prenatal care, medical and mental commorbidities
[b]Unknown Frequency pregnancies that had no self-reported cannabis use but tested positive on urine toxicology

© 2024 Avalos LA et al. *JAMA Network Open.*

eAppendix. Ascertainment of Maternal Prenatal Substance Use

As part of standard prenatal care at KPNC, patients are universally screened for prenatal substance use by both self-report (via a self-administered questionnaire) and urine toxicology tests to which they provide consent at entrance into care. Of those individuals with continuous health plan membership in the year prior to pregnancy through delivery and attendance at a minimum of one prenatal care visit, 93.0% were screened by self-report and urine test for prenatal cannabis use.

Maternal prenatal cannabis use was defined as self-reported cannabis use since becoming pregnant and/or a positive urine toxicology test at entrance to prenatal care. Results obtained from the first urine test during pregnancy ordered by OB/GYN department were used. If no urine test was ordered by OB/GYN department, results from the test closest to 8 weeks gestation were used. Cannabis toxicology screening tests were performed on a Beckman Coulter AU680 chemistry analyzer using the Emit II Plus Cannabinoid Assay from Siemens with a cutoff of 45ng/mL. Confirmatory testing for the presence of the cannabis metabolite, 11-nor-9-carboxy-delta 9- THC, was performed by liquid chromatography-tandem mass spectrometry for all positive immunoassay results. The confirmation test methodology was LC-MS/MS on a triple quadrupole system with a cutoff for positivity of 15ng/mL.

Prenatal substance use was defined as the following:

Variable	Definition
Prenatal alcohol use	• Self-report for prenatal alcohol use at entrance to prenatal care<br>• Toxicology for ethanol at entrance to prenatal care[1]
Prenatal opioid use	• Self-report for prenatal heroin, methadone, or buprenorphine use at entrance to prenatal care<br>• Toxicology for 6-monoacetylmorphine (6-MAM, heroin), morphine, codeine, hydrocodone, norhydrocodone, hydromorphone, oxycodone, nor oxycodone, or oxymorphone at entrance to prenatal care[1]<br>• Opioid prescription filled during pregnancy before first prenatal visit date or before pregnancy with supply lasting past pregnancy start date
Prenatal stimulant use	• Self-report for prenatal cocaine, crack, or methamphetamine use at entrance to prenatal care<br>• Toxicology for amphetamine, methamphetamine, MDMA (3,4-methylenedioxymethamphetamine) or MDA (3,4-methylenedioxyamphetamine) at entrance to prenatal care[1]<br>• Amphetamine prescription filled during pregnancy before first prenatal visit date or before pregnancy with supply lasting past pregnancy start date
Prenatal nicotine use	• Self-report for prenatal nicotine use at entrance to prenatal care<br>• Self-report for tobacco smoking during pregnancy before first prenatal visit date<br>• Nicotine replacement therapy prescription filled during pregnancy before first prenatal visit date or before pregnancy with supply lasting past pregnancy start date
Prenatal anxiety or sleep medication use	• Self-report for prenatal sleep or anxiety medication use at entrance to prenatal care<br>• Toxicology for benzodiazepines or barbiturates at entrance to prenatal care[1]

© 2024 Avalos LA et al. *JAMA Network Open.*

	• Anxiolytics or sedative prescription filled during pregnancy before first prenatal visit date or before pregnancy with supply lasting past pregnancy start date

[1]Results from the first test urine test during pregnancy ordered by OB/GYN department. If no urine test was ordered by OB/GYN department, results from the test closest to 8 weeks gestation were used.

Self-reported prenatal substance use

Items from KPNC Early Start Prenatal Screening Questionnaire (revised 2014) used in this analysis:

In general, how often have you used the following **since pregnancy**:

1. Alcohol (wine, beer, liquor, etc.)
2. Nicotine (cigarettes, Nicorette gum, chewing tobacco, nicotine replacement therapy [NRT])
3. Sleep medication (Trazodone, Ambien, Restoril, etc.)
4. Pain medication (Vicodin, Norco, Oxycontin, Percocet, Codeine, etc.)
5. Anxiety medication (Valium, Xanax, Ativan, etc.)
6. Marijuana
7. Cocaine or Crack
8. Methamphetamine (Speed, Crank, Ecstasy, Ice, etc.)
9. Heroin
10. Other street drugs, please specify:
11. Methadone or Buprenorphine (Suboxone or Subutex)

Response options: Never, Monthly or Less, Weekly, Daily

Self-reported nicotine use was also assessed from EHR data on current tobacco use; KPNC patients are routinely asked about current tobacco use by medical assistants taking vital signs at the start of primary care visits.

Prenatal urine toxicology testing

Alcohol: Alcohol screening tests were performed on a Beckman Coulter AU680 chemistry analyzer using the Emit II Plus Ethyl Alcohol Assay from Siemens with a cutoff of 10 mg/dL. Confirmatory testing for the presence of ethanol was performed by gas chromatography-mass spectrometry for all positive immunoassay results, also with a positive cutoff of 10 mg/dL.

Amphetamine/Methamphetamine: Amphetamine screening tests were performed on a Beckman Coulter AU680 chemistry analyzer using the DRI Amphetamines Assay with a cutoff of 500 ng/mL. Confirmatory testing for the presence of amphetamine species was performed by liquid chromatography-tandem mass spectrometry for all positive immunoassay results. Amphetamine species detected by the confirmation assay include amphetamine, methamphetamine, MDMA, and MDA. All species have a positive cutoff of 250 ng/mL.

Barbiturates: Barbiturate screening tests were performed on a Beckman Coulter AU680 chemistry analyzer using the Emit II Plus Barbiturate Assay from Siemens with a cutoff of 180 ng/mL. Confirmatory testing for the presence of barbiturate species was performed by liquid chromatography-

© 2024 Avalos LA et al. *JAMA Network Open.*

tandem mass spectrometry for all positive immunoassay results. Barbiturate species detected by the confirmation assay include butalbital and phenobarbital. All species have a positive cutoff of 100 ng/mL.

Benzodiazepines: Benzodiazepine screening tests were performed on a Beckman Coulter AU680 chemistry analyzer using the CEDIA Benzodiazepine Assay from Thermo Fisher Scientific with a cutoff of 200 ng/mL. This assay incorporates beta-glucuronidase treatment to detect total benzodiazepine species for those that have glucuronidated metabolites. Confirmatory testing for the presence of benzodiazepine species was performed by liquid chromatography-tandem mass spectrometry for all positive immunoassay results. Benzodiazepine species detected by the confirmation assay include alprazolam/alpha-hydroxyalprazolam, clonazepam/7-aminoclonazepam, flunitrazepam/7-aminoflunitrazepam, lorazepam, nordiazepam, oxazepam, temazepam, alpha-hydroxytriazolam, and zolpidem. All species have a positive cutoff of 25 ng/mL except for zolpidem which has a positive cutoff of 2.5 ng/mL.

Cocaine: Cocaine screening tests were performed on a Beckman Coulter AU680 chemistry analyzer using the Emit II Plus Cocaine Metabolite Assay which detects the cocaine metabolite benzoylecgonine with a positive cutoff of 150 ng/mL.
Opioids: Opiate screening tests were performed on a Confirmatory testing for the presence of benzoylecgonine was performed by liquid chromatography- tandem mass spectrometry for all positive immunoassay results. The positive cutoff for the confirmation assay was 100 ng/mL.

Beckman Coulter AU680 chemistry analyzer using the Emit II Plus Opiate 300 Assay from Siemens with a cutoff of 300 ng/mL. Confirmatory testing for the presence of opiate species was performed by liquid chromatography- tandem mass spectrometry for all positive immunoassay results. Opiate species detected by the confirmation assay include morphine, 6-monoacetylmorphine (6-MAM), codeine, hydrocodone, norhydrocodone, hydromorphone, oxycodone, noroxycodone, and oxymorphone. All species have a positive cutoff of 50 ng/mL except for 6-MAM which has a positive cutoff of 10 ng/mL.

© 2024 Avalos LA et al. *JAMA Network Open.*

eTable. Adjusted Hazard Ratios for Associations Between Maternal Prenatal Cannabis Use and ASD, Stratified by Infant Sex (N=178,948)

	**Male (n=91,876)**			**Female (n=87,072)**		
	ASD			ASD		
	Yes<br>n (%)<br>5,010 (5.5)	No<br>n (%)<br>86,866 (94.5)	HR[a] (95% CI)	Yes<br>n (%)<br>1,453 (1.7)	No<br>n (%)<br>85,619 (98.3)	HR[a] (95% CI)
Prenatal Cannabis Use						
No	4,734 (5.4)	82,801 (94.6)	1.00 (Ref)	1,358 (1.6)	81,569 (98.4)	1.00 (Ref)
Yes	276 (6.4)	4,065 (93.6)	1.01 (0.77- 1.32)	95 (2.3)	4,050 (97.7)	1.19 (0.77- 1.85)
Frequency of Cannabis use						
None	4,734 (5.4)	82,801 (94.6)	1.00 (Ref)	1,358 (1.6	81,569 (98.4)	1.00 (Ref)
Monthly or less	54 (5.4)	949 (94.6)	0.57 (0.31- 1.04)	16 (1.6)	984 (98.4)	1.18 (0.55- 2.50)
Weekly	31 (6.7)	430 (93.3)	0.74 (0.38- 1.44)	12 (2.6)	445 (97.4)	0.86 (0.24- 3.07)
Daily	29 (7.5)	360 (92.5)	1.51 (0.79- 2.87)	7 (2.0)	345 (98.0)	1.70 (0.40- 7.16)
Unknown Frequency[b]	162 (6.5)	2,326 (93.5)	1.13 (0.80- 1.60)	60 (2.6)	2,276 (97.4)	1.19 (0.70- 2.00)

[a]Adjusted for demographics, other non-cannabis substance exposure (alcohol, nicotine, opioids, anxiety/sleep medication, and stimulants), prenatal care utilization (Kotelchuck month of initiation index), medical and mental health comorbidities (asthma, diabetes mellitus, nausea/vomiting during pregnancy, mood/anxiety disorders, other psychiatric disorders, substance use disorders, antidepressant use, chronic pain).

[b] Unknown Frequency includes pregnancies that had no self-reported cannabis use but tested positive on urine toxicology.

© 2024 Avalos LA et al. *JAMA Network Open.*
